# Polysaccharide-Based Micro- and Nanosized Drug Delivery Systems for Potential Application in the Pediatric Dentistry

**DOI:** 10.3390/polym13193342

**Published:** 2021-09-29

**Authors:** Plamen Katsarov, Maria Shindova, Paolina Lukova, Ani Belcheva, Cédric Delattre, Bissera Pilicheva

**Affiliations:** 1 Department of Pharmaceutical Sciences, Faculty of Pharmacy, Medical University of Plovdiv, 4002 Plovdiv, Bulgaria; bisera.pilicheva@mu-plovdiv.bg; 2 Research Institute at Medical University of Plovdiv (RIMU), Vasil Aprilov Str. 15A, 4002 Plovdiv, Bulgaria; 3 Department of Paediatric Dentistry, Faculty of Dental Medicine, Medical University of Plovdiv, 4002 Plovdiv, Bulgaria; mariya.shindova@mu-plovdiv.bg (M.S.); ani.belcheva@mu-plovdiv.bg (A.B.); 4 Department of Pharmacognosy and Pharmaceutical Chemistry, Faculty of Pharmacy, Medical University of Plovdiv, 4002 Plovdiv, Bulgaria; paolina.lukova@mu-plovdiv.bg; 5 Université Clermont Auvergne, Clermont Auvergne INP, CNRS, Institut Pascal, 63000 Clermont-Ferrand, France; cedric.delattre@uca.fr; 6 Institut Universitaire de France (IUF), 75005 Paris, France

**Keywords:** microparticles, nanoparticles, drug delivery systems, natural polymers, polysaccharides, chitosan, alginate, pectin, dextran, pediatric dentistry

## Abstract

The intensive development of micro- and nanotechnologies in recent years has offered a wide horizon of new possibilities for drug delivery in dentistry. The use of polymeric drug carriers turned out to be a very successful technique for formulating micro- and nanoparticles with controlled or targeted drug release in the oral cavity. Such innovative strategies have the potential to provide an improved therapeutic approach to prevention and treatment of various oral diseases not only for adults, but also in the pediatric dental practice. Due to their biocompatibility, biotolerance and biodegradability, naturally occurring polysaccharides like chitosan, alginate, pectin, dextran, starch, etc., are among the most preferred materials for preparation of micro- and nano-devices for drug delivery, offering simple particle-forming characteristics and easily tunable properties of the formulated structures. Their low immunogenicity and low toxicity provide an advantage over most synthetic polymers for the development of pediatric formulations. This review is focused on micro- and nanoscale polysaccharide biomaterials as dental drug carriers, with an emphasis on their potential application in pediatric dentistry.

## 1. Introduction

Pediatric dentistry includes diagnostics, prevention and treatment of specific diseases and oral conditions associated with pediatric dental patients [1,2]. The most common dental diseases during the period of childhood are dental caries and diseases of periodontium. The main aim of contemporary pediatric dentistry is risk assessment of oral diseases and early diagnostics, individual preventive program development and initial treatment [3,4]. The unquenchable thirst for gathering new knowledge and striving for scientific development result in exploitation of the innovations in pediatric dentistry as well. 

The rapid development of micro- and nanotechnologies in recent years and their gradual implementation in dentistry provoke more researchers to focus on the development of novel polymer-based therapeutic systems and their imposition in personalized oral treatment. Such systems provide not only new options for prevention and treatment of oral diseases but also improvement of the properties of the materials used for operative, endodontic and periodontal treatment for children [5,6]. Micro- and nanoparticles based on polymers offer various advantages as drug delivery systems. By incorporating the drug in a polymer matrix (carrier) a sustained drug release can be accomplished [7,8,9,10]. Thereby, a desired therapeutic effect can be achieved by administering reduced doses of the drug. At the same time the lower risk of drug side effects and toxicity can provide a great advantage in the development of therapeutics for children. Polymer drug delivery systems can also be designed for targeted therapy, enabling the drug to be directed to a desired location in the oral cavity [6,11]. Furthermore, due to their small size, nanoparticles can reach regions that may be inaccessible to other delivery systems, such as the deep periodontal pockets [12]. Nanosizing favors the absorption and bioavailability of many drugs, leading to a reduction in the drug dose and the frequency of its administration [13,14,15]. The different types of polymer micro- and nanostructures, some of their preparation methods and advantages as drug carriers are presented in Table 1.

**Figure 1:**
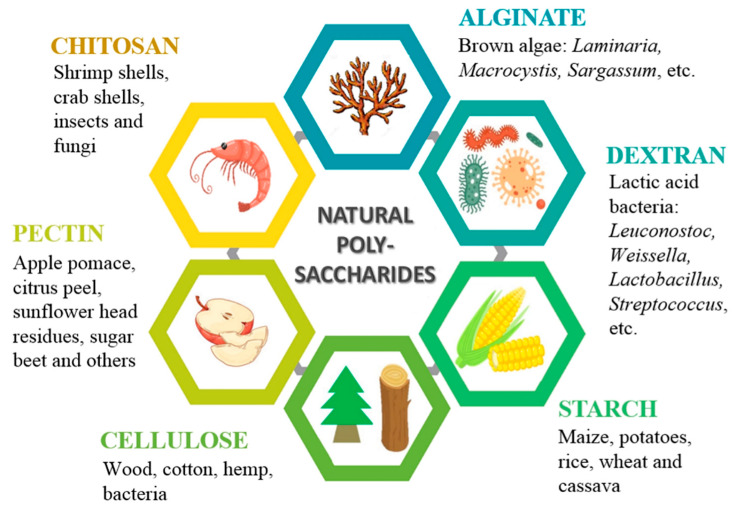
Main natural polysaccharides from various sources, which can be used as drug carriers.

Over the years, different drug carriers have been investigated and tested for drug delivery and targeting in the oral cavity. Natural polymers like polysaccharides are among the most preferred materials in dental practice, especially for the development of pediatric formulations, due to their biocompatibility, biotolerance and biodegradability. They display low or no toxicity, which provide an advantage over most synthetic polymers [16]. Naturally occurring polysaccharides such as chitosan, alginate, pectin, dextran, starch, etc. (Figure 1) are being widely exploited in the medical and dental practice, providing a range of different applications [17,18,19]. They are often used in the preparation of biodegradable micro- and nano-devices for drug delivery, offering simple particle-forming characteristics and easily tunable properties of the formulated structures [20,21]. 

Moreover, most polysaccharides have good adhesion to mucous membranes and enamel surfaces, which is a prerequisite for longer retention at the site of application and prolonged delivery of drugs in the oral cavity [22,23]. There are many examples in the literature reporting the successful use of polysaccharides as micro- and nano drug carriers and outlining their promising application in dentistry [6,9,24,25,26]. However, the available information so far has not been summarized, which determines the urge for performing a thorough review on that issue.

The aim of the current work was to present an overview of the advantageous applications of naturally occurring polysaccharides as micro- and nano-drug carriers in dentistry. Furthermore, a special focus was put on the pediatric practice and the most used treatment approaches related to polysaccharide-based drug delivery systems in children oral care.

## 2. Materials and Methods

The review article is based on the literature found in the databases of PubMed, Web of science and Science Direct. The performed survey was within the year interval 2000–2020, and 158 references were selected for the review. The choice of publications was made on the basis of the relevance of the publications to the topic, the research methodology, the research results and the year of publication. The cited publications include systematic reviews, research articles, book chapters and meta-analysis. 

## 3. Natural Polysaccharide Drug Carriers in Dentistry

Polysaccharides are a large group of biological substances, which are composed of monosaccharides (sugars) linked together by O-glycosidic linkages. Their properties depend mainly on their monosaccharide composition, linkages and molecular weight [27]. 

Natural polysaccharides are widely distributed in nature and can be obtained from renewable resources, like plants, algae, fungi, animals and microorganisms (Figure 1). That makes them affordable and cost-effective materials for various medical and dental applications [22,28,29]. Moreover, their properties enable relatively easy and reproducible production of drug formulations by applying already well-studied production methods such as spray drying, emulsion technique, coacervation, polymerization, etc. [26,30,31,32]. By choosing the right production parameters, polysaccharide-based micro- and nanoparticle can be developed with desired size, high yield, high drug encapsulation efficiency and controlled drug release, which could make them promising drug delivery systems in the dental practice.

### 3.1. Chitosan

**Figure 2:**
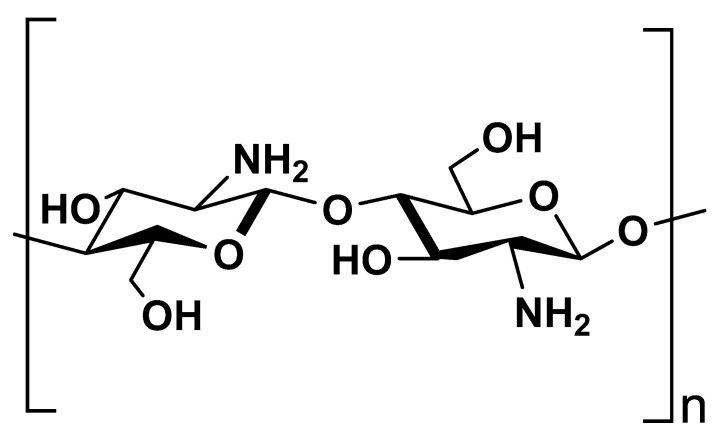
Chemical structure of chitosan.

Chitosan is a natural polymer derived from chitin through deacetylation. It is chemically comprised of *N*-acetylglucosamine and glucosamine copolymer units (Figure 2) [33,34]. Due to its biocompatibility, biodegradability and non-toxic properties, chitosan is one of the most extensively exploited polymers as biomaterial in the medical and dental practice [35,36,37].

Moreover, chitosan possesses a strong antiplaque activity. It causes destruction of the bacterial cells by promoting displacement of Ca^2+^ of the anionic sites of the cellular membrane [38]. Chitosan has been proven effective against oral bacteria such as *Porphyronomas gingivalis*, *Prevotella intermedia* and *Actinobacillus actinomycetemcomitans* and other pathogens [39,40]. Aliasghari et al. reported an inhibitory effect of chitosan nanoparticles against *S. salivarius* and *S. sobrinus* at a concentration of 0.625 mg/mL. Chitosan nanoparticles at a concentration of 5 mg/mL reduced up to 93.4% of the biofilm formation of the bacteria *S. mutans*, which played an important role in the pathogenesis of dental caries [41]. Another study showed the promising antibacterial activity of copper-loaded chitosan nanoparticles against *S. mutans*, which was compared to that of oral antimicrobial agents such as chlorhexidine and cetylpyridinium chloride [42]. It was believed that chitosan interacted with tooth hydroxyapatite and bacterial cell wall, enhancing the adherence of copper to the tooth surface. The presence of cationic amino groups in its molecule allows chitosan to be adsorbed through electrostatic forces onto materials with strong negative zeta potential, such as the tooth enamel [43]. Chitosan nanoparticles have also been proposed as a perspective coating material for titanium dental implants. Divakar et al. have determined an enhanced antimicrobial activity of chitosan conjugated silver nanoparticles against dental implant pathogens. They have concluded that the formulated chitosan nanoparticles are a good option to provide strong antibacterial effect, being at the same time biocompatible and not causing cell cytotoxicity [44].

Chitosan has been widely used for developing drug delivery systems for application in the oral mucosa and gingiva because of its excellent mucoadhesive properties. This characteristic is attributed to the cationic nature of the polymer, which helps forming ionic bonds with the negatively charged mucin in the mucus membranes [45]. Providing longer mucosal residence time is essential for treating local inflammatory conditions in the oral cavity when a prolonged therapeutic effect at the site of application is desired. Conventional antimicrobial formulations for the mouth, such as toothpaste and mouthwash, have short duration of action and very low penetration into the periodontal pocket due to the rapid clearance of the administered biomolecules [24]. An ideal local delivery system should be able to deliver antimicrobial drugs in a controlled manner with good retention at the application site. In this regard the mucoadhesive chitosan micro- and nanoparticles are often preferred systems that can deliver therapeutic molecules for treatment of gingivitis and periodontitis and release them in a sustained manner [46]. Braga et al., for example, formulated mucoadhesive chitosan microspheres loaded with ketoprofen for use in the treatment of periodontal disease [30]. By using chitosan for microencapsulation they achieved 4.6 times longer release of ketoprofen from the microparticles (t_50_: 36 h) compared to the free drug (t_50_: 7.84 h). In a study by Dias et al., a novel injectable formulation containing chitosan microparticles loaded with ornidazole was proposed for the treatment of periodontitis [47]. The authors performed an in vitro adhesion test on sheep cheek mucosa and proved high bioadhesion of the obtained chitosan particles. They observed improved adhesion with the increase in the polymer concentration, which was attributed to the availability of a greater number of polymer chains for interaction with the mucus. The ornidazole-loaded microspheres showed diffusion controlled sustained drug release for up to 5 days. Govender et al. have reported the application of chitosan microspheres for delivery of tetracycline to the periodontal pocket [48]. The authors have employed a statistical experimental design in order to formulate a microspheres preparation with maximum bioadhesiveness and controlled drug release. Contrary to expectations, they found that chitosan concentration had a negative effect on bioadhesion. Probably coiling of the polymer molecules occurred at high concentrations, which limited the polymer chain’s flexibility. Microspheres, obtained at lower chitosan concentration, were thought to have looser structure, which provided more space for the polymer chains to entangle with the mucin.

**Figure 3:**
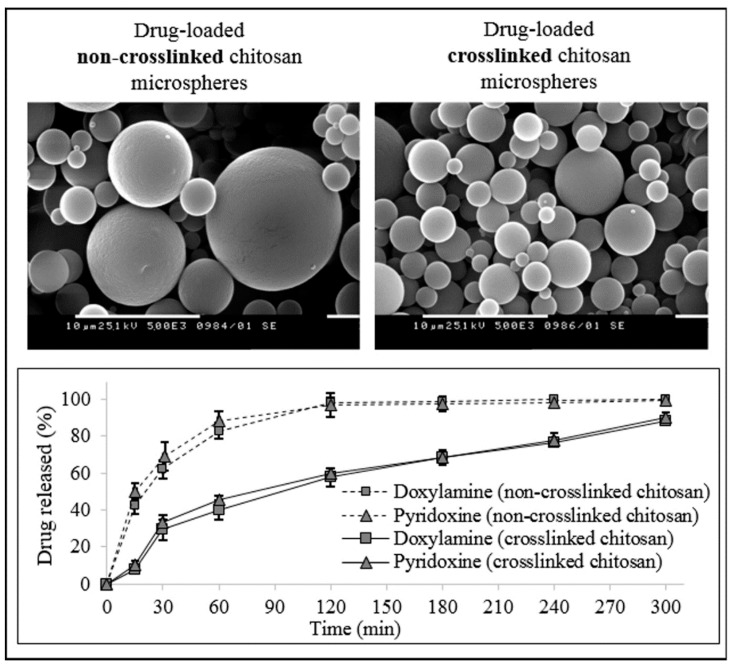
Scanning electron micrographs and drug release profiles of spray-dried, non-crosslinked and crosslinked chitosan-based microparticles, loaded with model drugs—doxylamine and pyridoxine.

Drug release from chitosan particles could be controlled by crosslinking the polymer matrix, which has been investigated in one of our previous studies (Figure 3) [49]. 

The crosslinking agents for chitosan can ionically or covalently bind the polymer chains. The former includes substances which have a negative charge and create ionic bridges between the positively charged chitosan chains. They can be multivalent metal anions: Mo (VI), Pt (II) or molecules often loaded with phosphate groups, such as tripolyphosphate sodium (TPP) [50]. TPP was used by Suresh et al. for crosslinking of chitosan in order to formulate microparticles for localized controlled release of metronidazole following insertion into and/or around the periodontal pocket [25]. Their aim was to maintain an effective drug concentration at the periodontal site over an extended period of time, limiting the distribution of the drug to other body organs and decreasing its side effects. The authors showed that with the increase in the TPP a prolonged drug release was achieved up to 24 h. Furthermore, the used crosslinking agent did not negatively affect the polymer bioadhesion. On the contrary, an increased bioadhesion was registered with an increase in crosslinking. This was explained with more sites on the negatively charged sialic acid residues of mucin, remaining available for interaction with the positively charged drug.

Although some modification of the polymeric carrier can be achieved with negatively charged molecules or ions, substances that form strong covalent bonds with its chains are more effective in crosslinking chitosan. The molecules of these crosslinking agents must have at least two free functional groups. Dialdehydes (glutaraldehyde, glyceraldehyde, glutaric acid, etc.) are the most widely used agents for this purpose. They chemically crosslink the polymer by binding to the free amino groups of chitosan, forming stable imine structures [51]. Pichayakorn et al. have investigated the effects of different concentrations of glutaraldehyde and cross-linking time on the characteristics of chitosan microparticles containing metronidazole for periodontitis treatment [26]. The results indicated that the optimal conditions for microparticles with a high percentage of entrapped drug and preferable release profile were 1:1 drug:chitosan ratio, 5% glutaraldehyde based on chitosan solution and 30 min cross-linking time. The authors have proposed a hydrogel formulation containing the obtained particles as a promising drug delivery system with prolonged release of metronidazole to be clinically used for periodontitis. Although glutaraldehyde has been established as very effective in crosslinking chitosan, the safety of its use and the risk of toxicity should be taken into consideration.

The examples given so far confirm the widespread use of chitosan as a drug carrier and its significant potential for modified or targeted drug delivery in the oral cavity (Table 2). Although it has already been extensively studied as a material for creating micro- and nano-drug carriers, the constant investigation of new possibilities for its functionalization stimulates researchers to continue their research on it.

### 3.2. Sodium Alginate 

**Figure 4:**
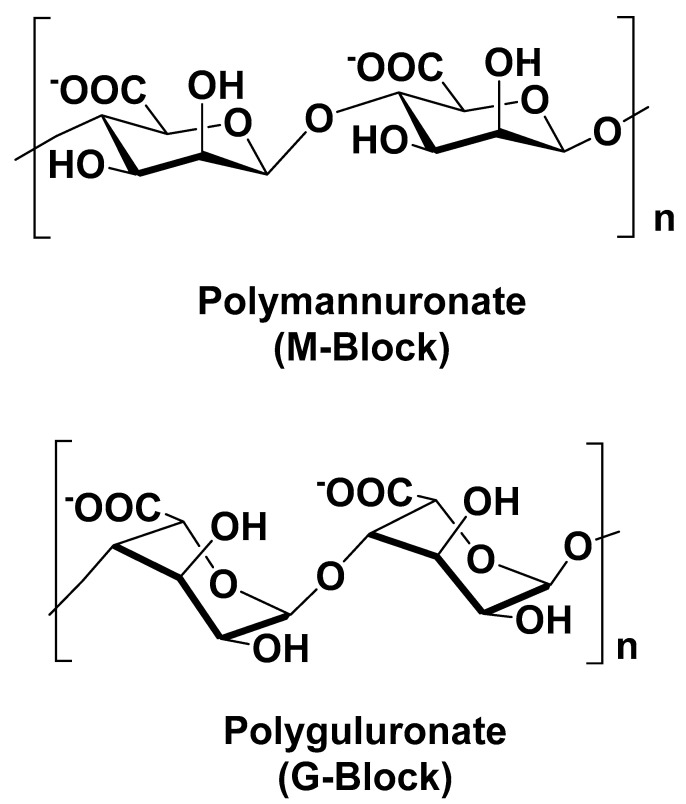
Main chemical structures of alginate blocks.

Alginates are natural water-soluble polysaccharides extracted from the cell wall of various species of brown algae. They consist of linear copolymers of *β*–(1,4) linked D–mannuronic acid (M) and *β*–(1,4)-linked L–guluronic acid (G) units (Figure 4). Mannuronic and guluronic blocks can be arranged in homogenous (poly-G, poly-M) or heterogenous (MG) patterns [28]. 

**Figure 5:**
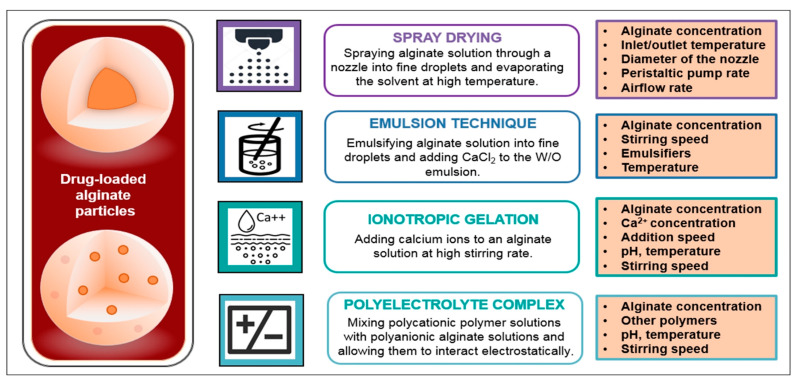
Main methods for formulation of drug-loaded alginate particles and the basic parameters, affecting the production process.

Like chitosan, sodium alginate is non-toxic, biodegradable and biocompatible in the oral environment, which makes it another valuable biopolymer for application in the dental practice. Due to their hydrophilic nature, elasticity and low cost, alginates are the most widely used impression materials in dentistry [59,60,61]. However, their potential as carriers for oral drug delivery also should not be neglected. The main methods for preparation of alginate particles as drug delivery systems are presented in Figure 5 [20,62]. For example, alginate-based microbeads were developed as promising local chlorhexidine releasing devices for periodontal therapy [63]. The addition of the active substance to the alginate solution led to an ionic interaction with the polymer and initiated a gelling process. This required the production process to go through two stages—obtaining drug-free polymer microparticles and their subsequent drug loading by diffusion of chlorhexidine into them. The reported results indicated that the type of production method significantly affected the size of the obtained microstructures. By emulsifying an alginate solution in an oil phase and gelling with calcium ions (internal gellation), an average particle size of 100–400 µm was achieved. Smaller structures with a size of 20–70 μm were obtained by ultrasonic spray technique. According to this method, alginate solution was dispersed by ultrasonic energy into CaCl_2_-solution (external gelation) using nozzle’s tip with a needle of 0.5 mm diameter. The release of chlorhexidine from the formulated particles was determined in vitro using artificial saliva and the results showed that alginate-based beads had comparable releasing characteristics as clinically approved systems.

Alginate in combination with chitosan has been proposed as an effective encapsulation agent for minocycline, an antibiotic which is typically used for treatment of periodontal diseases [64]. In a study by Park et al., alginate-chitosan microspheres loaded with 10% minocycline were prepared by ionotropic gelation method [65]. The particles were designed as a biodegradable device for implantation in the periodontal pocket, providing drug concentrations in the gingival fluid for seven days. The results indicated a substantial activity of the novel formulation against pathogenic bacteria, such as *Prevotella intermedia,* causing periodontitis.

Ferraz et al. developed injectable microparticles for delivery of antibiotics, used for periodontitis treatment—amoxicillin, amoxicillin with clavulanic acid, and erythromycin [66]. Microspheres with a median diameter of 450 µm were prepared through an extrusion methodology, proposed by the authors, using sodium alginate and hydroxyapatite in a ratio 4:1 *w*/*w*, and a 0.1M solution of CaCl_2_ as a crosslinking agent. The obtained particles showed fast initial release followed by a sustained release of the incorporated antibiotics, which outlined them as good alternatives for a delivery system of the studied drugs. Furthermore, they expressed osteoconductive properties, enhancing bone regeneration while treating periodontitis.

Moreover, microencapsulation of cells using alginate has been studied as a potential approach for bone-tissue engineering in the regenerative dentistry. Alipour et al. have cultured human dental pulp stem cells in alginate-gelatin microcapsules. The results demonstrated a promoted cell proliferation and osteogenic differentiation [67].

### 3.3. Pectin

**Figure 6:**
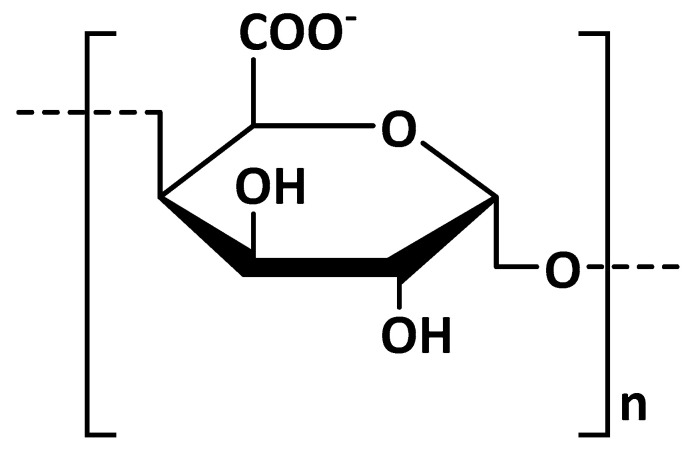
Main chemical structure of pectin made up of galacturonic acid block (polygalacturonic acid).

Pectin is an anionic polymer mainly extracted from citrus or apple fruits, composed of D–galacturonic acid and L–rhamnose units (Figure 6). It is a non-toxic natural polysaccharide, often used in food and drinks as a thickening and gelling agent [68,69]. Pectin has demonstrated a beneficial effect against emanel erosion caused by acids, which is a major factor for tooth decay. Adding pectin to acidic soft drinks has been established as an important approach to reduce dental erosion [70].

As a drug carrier, pectin has demonstrated bioadhesive properties to mucin and mucous membranes, as well as adsorption to enamel surfaces [71]. Several studies reported the formulation of pectin nano- or microstructures encapsulating active substances [11,72,73]. Although different methods can be applied in order to develop such polymer particles like emulsion-based techniques, coacervation or spray drying, the basis for the formation of micro- and nanostructures is usually ionotropic gelation of pectin [74,75,76]. Similar to alginate, pectin forms a gel structure in the presence of calcium, zinc or copper ions, which is due to the formation of strong ionic bonds between the cations and the galacturonic acid of the polymer [77,78]. The rapid swelling and dissolving of pectin in the saliva is usually marked as a disadvantage for the polymer in terms of producing long-acting delivery systems. Esposito et al. have emphasized on the need of crosslinking procedures in the formulation of drug-loaded pectin microparticles in order to reduce polymer dissolution and prolong the drug release [72]. They evaluated the encapsulation in pectin microspheres of two antibiotics—metronidazol and tetracycline, which can be applied in the treatment of periodontal diseases. Calcium chloride was used as an ionic crosslinker in order to modify the rapid swelling and solubility in water of the pectin microparticles. It has been demonstrated by means of particle size modification and hardening procedures that pectin microcapsules with desired morphological and dimensional characteristics can be formulated as perspective systems intended for controlled release of drugs.

An interesting alternative to the divalent ions for the gelation of pectin has been proposed by some authors, who used chlorhexidine not only as a dental antiseptic, but also as a reagent in the formation process of the polymer particles. Lasco et al. formulated chlorhexidine-loaded pectin microparticles using the active substance as a cross-linking agent for the polymer [73]. They reported that the drug-pectin interactions were so strong that the release of the drug was highly limited. Zinc ions were included in the optimal microparticle formulation for chlorhexidine delivery. They competitively interacted with pectin, limiting the formation of drug-polymer bounds, which provided a weaker structure of the gel network and allowed an improvement of the drug release.

Another application of pectin in the development of drug delivery systems for the dental practice is related to its negative charge and the ability to increase the stability of liposomal structures in the oral cavity. Pistone et al. have demonstrated that the surface charge of the nanostructures was of great importance for both their stability in salivary environment and bioadhesion [11]. The authors have formulated polysaccharide-coated liposomes for application as nano-sized delivery systems addressed to teeth. Although they determined that chitosan provided the highest in vitro adsorption onto hydroxyapatite in the presence of artificial saliva, its positively charged liposomes showed instability due to significant aggregation. The authors emphasized the stabilizing effect of pectin as a coating polymer. The negatively charged pectin-coated liposomes showed high stability without aggregation in the artificial saliva and were suggested as promising formulations to be used as a tooth adhesive nanosystem, providing improved treatment of tooth ailments. Similar conclusions were reported by other researchers, who have also studied the potential use of pectin in the formulation of liposomes [79,80,81].

### 3.4. Dextran

**Figure 7:**
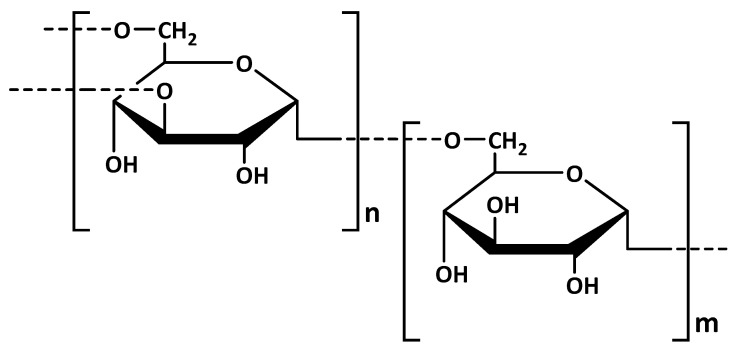
Chemical structure of dextran.

Dextran is a complex branched polysaccharide, synthesized by lactic acid bacteria or their enzymes in the presence of sucrose. The polymer linear chain consists of D–glucoses linked by *α*-(1→6) bonds with possible branches of D–glucoses linked by *α*-(1→4), *α*-(1→3), or *α*-(1→2) bonds (Figure 7) [29]. Dextran has been considered as a promising polymer carrier candidate for a wide variety of therapeutic agents due to its physico-chemical properties and physiological acceptance [82].

Wu et al. used dextran for the development of an intrapocket delivery system of minocycline for periodontitis treatment. They applied ion pairing/complexation technique to formulate minocycline-calcium-dextran sulfate complex microparticles with high encapsulation efficiency (97%) and high drug loading (45%). The obtained delivery systems demonstrated potent antimicrobial effects against *Streptococcus mutans* and *Aggregatibacter actinomycetemcomitans*. The in vitro studies showed sustained release of minocycline for at least 9 days at pH 7.4 and 18 days at pH 6.4 in phosphate-buffered saline [10].

Dextran in combination with poly-(lactic-co-glycolic acid) has been utilized for formulating microparticles, loaded with interleukin 1 receptor antagonist (*IL-1ra*). The results suggested that the developed microspheres were excellent candidates for periodontitis treatment, effectively inhibiting the gene expression of pro-inflammatory factors induced by *IL-1β* in human gingival fibroblasts [83].

Some studies define dextran as a suitable microcarrier for gene delivery of bone regeneration growth factors in patients, needing dental implant treatments to restore oral functions [84,85,86]. A novel microparticle formulation for periodontal tissue regeneration, based on dextran, was suggested in 2005 by Chen et al. [84]. The authors encapsulated recombinant human bone morphogenetic protein-2 (*rhBMP2*) with dextran using double-phase emulsified condensation polymerization. *RhBMP2* is a potent osteoinductive growth factor, inducing bone formation by stimulating the differentiation of mesenchymal cells into chrondroblasts and osteoblasts. It has been commercially available in orthopaedics, but it has also been applied to improve bone regeneration in challenging cases requiring dental implant treatment [87]. However, complications related to an initially high dosage for maintaining an effective physiological concentration at the defect site have been reported, which determined the need of a polymer carrier like dextran in order to achieve a protein delivery in the oral tissue in a sustained manner. By encapsulating *rhBMP2* into dextran-based microspheres, Chen et al. demonstrated that equivalent therapeutic effect could be achieved with smaller quantity of *rhBMP2*. The formulated dextran microspheres showed high encapsulation efficiency (82%), long-term stability (6 months at storage below 4 °C) and prolonged retention both in vitro and in vivo.

The use of organic solvents for the formulation of polymer micro- and nanoparticles could become a critical issue, especially in pediatric dentistry, due to possible toxic effects. Avoiding such solvents is also essential to prevent potential damage and bioactivity loss of the encapsulated protein structures like the human bone morphogenetic protein (*BMP)* during particle preparation. These arguments led to the development of a modified dextran-based microcarrier for *rhBMP2* [85]. A dextran-based precursor was synthesized by substituting the polysaccharide hydroxyl groups with glycidyl methacrylate (*Dex-GMA*). The precursor was then used to formulate microspheres in an aqueous two phase system by polymerization of *Dex–GMA* emulsified in a poly-(ethylene glycol) solution. The obtained microspheres with *rhBMP2* were acceptable for injection particle size in the range from 10 to 60 µm in diameter, high encapsulation efficiency (86%) and in vitro sustained protein release (more than 60% of the drug were released in 20 days).

In a further attempt to accomplish functionalized modification of the *BMP* carriers and enhanced biological activity, dextran microspheres loaded with bone morphogenetic proteins were incorporated into a newly synthesized glycidyl methacrylated dextran/gelatin hydrogel scaffold [86].

A similar approach has been applied for locally controlled delivery of insulin-like growth factor-I (*IGF-I*) from dextran–co-gelatin microspheres [88]. *IGF-I* is a polypeptide growth factor, which plays a very important role in the biology of oro-dento-facial tissues and organs, including the development and regeneration of the periodontium [89]. In an aqueous solution, the positively charged protein could easily interact by polyionic complexation with negatively charged gelatin and thus be immobilized in the polymer matrix. In this case gelatin was preferred for cooperation with glycidyl methacrylate dextran (*Dex-GMA*). *IGF-I* incorporated dextran-gelatin delivery systems showed a significant biological effect on periodontal healing enhancement, which was attributed mainly to the nature of the microspheres that could provide proper drug protection, permeation enhancement and enzyme inhibition. Another advantage of the proposed system was the prolonged protein release (more than 28 days) at a relevantly constant rate after an initial burst effect.

At a later stage, dextran nanoparticles have been suggested as an alternative to the above described protein microcarriers. Composite glycidyl methacrylated dextran (*Dex-GMA*)/gelatin nanoparticles with mean diameter of 53.7 nm were formulated to deliver growth factors for periodontal regeneration, taking advantage of their small size and the possibility of better biodistribution as well as site- and cell-specific drug delivery [90].

### 3.5. Starch

**Figure 8:**
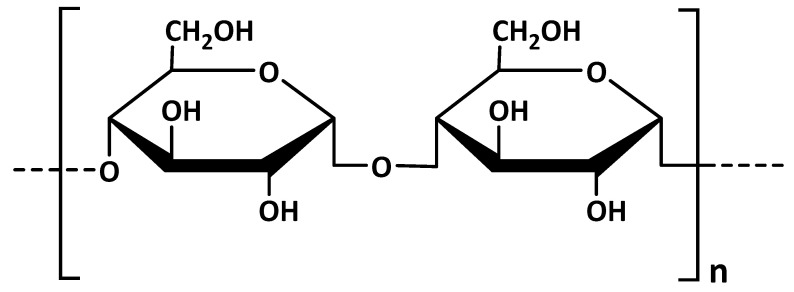
Main chemical structure of starch.

Starch is another biopolymer used in the development of micro- and nanocarriers for various medical and dental purposes. It is a polysaccharide consisting of anhydroglucose units linked together primarily through *α*–D–(1→4) glucosidic bonds (Figure 8). Its structure can be divided into two parts: amylose (linear structure of *α*-1,4 linked glucose units) and amylopectin (branched structure of *α*-1,4 chains linked by *α*-1,6 bonds) [91]. Starch has attracted attention due to its inherent biodegradability, annual renewability in nature and low material cost. It has also been included, although not so widely, in several drug delivery systems for dental practice [92,93,94,95].

The potentially harmful effects of many of the synthetic therapeutic agents used in the prevention and treatment of children’s tooth decay and periodontitis, necessitate the search for alternative approaches and the application of more gentle natural biomolecules. Rezapour et al. have proposed the use of curcumin for decreasing dental caries, formulating starch nanoparticles as its carriers [92]. Curcumin is a natural anti-inflammatory agent (produced by plants of the *Curcuma longa* species), which indirectly prevents the formation of biofilm and plaque on teeth, being active against the oral bacteria *Streptococcus mutans* [96]. The suggested starch-based nanostructures seemed to be a successful strategy for delivering the active substance in the oral cavity, overcoming limitations like curcumin poor solubility and bioavailability.

A more trivial approach against *Streptococcus mutans* was proposed by Costa One et al., who prepared, using spray drying technique, starch nanocapsules with chlorhexidine [93]. They tested in vitro the antimicrobial activity of the obtained nanoparticles and reported significant efficiency—90% cell death of *S. mutans* in artificial saliva. Moreover, a controlled drug release was observed, which allowed the application of the active substance at lower concentrations—reducing its side effects and at the same time preserving its therapeutic efficacy.

Encapsulation of chlorhexidine with starch has also been studied by Queiroz et al. [94]. The authors developed a polysaccharide-based film, containing the active substance, incorporated into nanoparticles. The proposed drug delivery system was produced through a simple, cheap and reproduceable process. A water/glycerol solution of starch was heated till gelatinization and after adding ethanol and chlorhexidine under stirring, a film was formulated, containing drug-loaded particles. The formation of nanoparticles was attributed to the starch precipitation with ethanol and subsequent absorption of the drug. The conducted in vitro drug release studies indicated that the delivery system could be active for more than 21 days.

Moreover, starch has been used as a carrier in the fabrication of metal nanoparticles with antibacterial activities. Kassaee et al. synthesized silver nanoparticles, stabilized by starch [95]. They performed γ-ray reduction of silver ions in aqueous starch solutions, deriving optimal parameters for the formation of particles with narrow size distribution and high production yield: 5 kGy γ -irradiation of a 2 × 10^−3^ M solution of AgNO_3_ containing 0.5% starch.

The presented examples prove that starch has great potential as a drug carrier in dental practice. Nevertheless, its use in pediatric dentistry should be approached with precaution, taking into account that starch itself may possess significant cariogenic effect [97]. The possible starch-caries issue rather makes starch not a polymer of first choice for use in pediatric dentistry.

### 3.6. Other Polysaccharides

Cellulose and its derivatives have been widely exploited as drug carriers for the formulation of micro- and nanostructures [98,99,100,101,102,103,104,105]. As early as 1983, attempts were made to achieve a sustained delivery of tetracycline into the periodontal pocket, developing a reservoir type of device made up of cellulose acetate [98]. The polymer systems released their drug load within 24 h by a diffusion mechanism. However, the formulations showed brittle physical properties and were not tested clinically. In more recent studies, cellulose was used rather as a material for impregnation of silver nanoparticles [99,100,101]. Lately, there has been an increased interest in bacterial cellulose and its use as a drug carrier in the dental practice [102,105].

Hyaluronic acid is a naturally occurring linear polysaccharide, which is a key element in the soft periodontal tissues, gingiva, and periodontal ligament [106]. In the field of dentistry it is mainly used in postoperative dental surgery to improve the healing process. Moreover, hyaluronic acid has recently been recognized as an adjuvant treatment for acute and chronic gingivitis and periodontitis. Various clinical trials have shown its anti-inflammatory, anti-oedematous and anti-bacterial effects against microorganisms present in subgingival plaque [106,107]. Hyaluronic acid has been well-studied for the development of drug-loaded micro- and nanoparticles, which makes it a promising candidate for a drug delivery carrier in the dental practice [108,109].

Curdlan is a β–(1→3)–D–glucan, produced mainly from *Alcaligenes faecalis*, but also from some *Rhizobium*, *Cellulomonas* and *Agrobacterium* strains. It is a high molecular weight polysaccharide (Mw > 2.0 × 10^6^ Da), forming a similar to starch structure [110]. Curdlan-based microspheres have been evaluated for drug targeting on mucosal tissues and for controlled release of active agents and vaccines [111]. There are also studies in the literature, reporting the successful utilization of the polysaccharide as a nano-sized carrier for intracellular siRNA delivery, for nanoencapsulation of curcumin and for green synthesis of silver nanoparticles [112,113,114]. These examples prove the potential of curdlan as a drug carrier with possible application in dental practice.

Studies indicated that xanthan gum showed acceptable bioadhesion in theperiodontal pocket and oral mucosa [115]. This polysaccharide has been used for developing a delivery system for targeted release of chlorhexidine and metronidazole [116,117].

Other natural polysaccharides, which have been investigated as micro- and nano-drug carriers are pullulan and fucoidan [118,119].

## 4. Applications of Polysaccharide Micro- and Nanoparticles in Pediatric Dentistry

**Figure 9:**
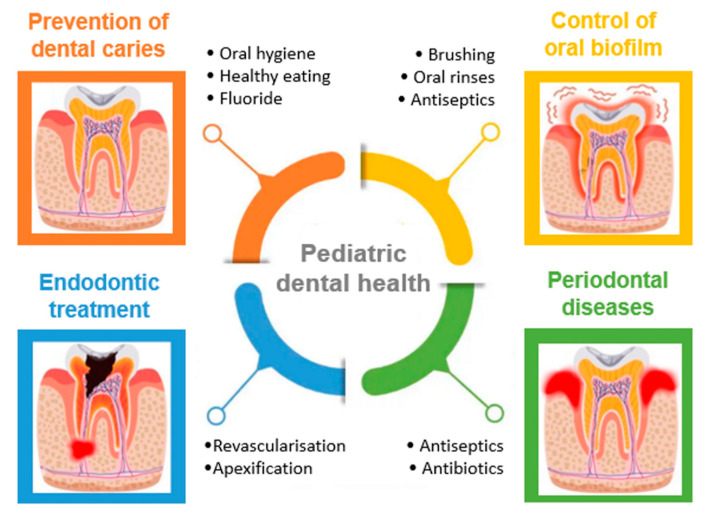
The most common diseases and oral conditions associated with pediatric dental patients.

The examples discussed so far clearly confirm the applicability of micro- and nano-drug carriers in dentistry. The introduction of such innovative therapeutics into the pediatric dental practice is a slow process that requires in-depth research on their safety and efficacy. However, polysaccharide-based drug delivery systems have the potential to become promising therapeutic approaches in the treatment of the most common dental conditions and diseases during childhood, such as: prevention of dental caries, control of oral biofilm, endodontic treatment and periodontitis (Figure 9).

### 4.1. Prevention of Dental Caries—Primary and Secondary Prevention

#### 4.1.1. Fluoride

Both professional and home-use applications are considered to be significantly effective in caries prevention with regard to fluoride topical methods [120,121,122]. Apart from the popular fluoride-containing products for professional use in the prevention of dental caries such as varnishes, gels, rinses, foam, etc., in recent years innovative products have been developed. During their first use, they are professionally applied and then they can be self-applied (for home use). The products are called fluoride-containing bioadhesive slow-release tablets in which the active agent is encapsulated into polymeric micro- or nanoparticles which ensure a prolonged release of fluoride as a delivery system. There are different places in the oral cavity where they could be applied—oral mucosa, hard dental tissue surfaces, steel bracket or wire arch, etc. [123,124]. In a recent clinical study, a similar mechanism of action was demonstrated by nanoparticles in the presence of sodium fluoride (NaF) as an active ingredient [54]. The aim of the team was to investigate the efficiency of biopolymers like chitosan, alginate, and pectin as a basis for bioadhesive and biocompatible nanoparticles loaded with fluoride for caries prevention. The results showed that pectin and alginate were able to form stable nanoparticles in an acidic environment similar to those during cariogenic attacks. However, chitosan as a carrier was the most effective polymer, ensuring continuous delivery of the caries protective agent. The results of an experimental study by Ebrahim et al. also supported the promising action of fluoride/chitosan nanoparticles [55]. Up to 2017, there was only one clinical study that reported the effect of the use of fluoride ions incorporated into bioadhesive fluoride tablets [125]. The review of the contemporary scientific literature provides insight and demonstrates numerous in vitro and ex vivo studies, investigating the succession of this therapeutic approach [126,127].

Along with nanosystems, microparticles using chitosan as a carrier and active agent NaF also showed potential for optimizing the release of fluoride ions and thereby improving its preventive action [56]. A series of in vitro and ex vivo studies are required to confirm the “in vivo” obtained results up to now and to enhance the clinical significance of oral care products containing nano and microparticles.

In addition to the direct effect of fluoride ions on enamel, the mineralizing effect, when slow-releasing fluoride chitosan-coated nanoparticles were used, was due to the increased fluoride concentration found on the tooth surface and higher resistance to the cleansing action of the salivary flow [54].

#### 4.1.2. Silver

Nanostructures called nano-silver fluoride systems have also been suggested as an option for caries preventive therapy and have been recently investigated in an in vitro study conducted by Targino [57]. Chitosan was used as a stabilizing agent and a carrier for silver nanoparticles and fluoride. The results of the study reported low toxicity and high efficiency in low doses of the newly investigated composition in comparison with chlorhexidine. Both active agents in the research (silver and fluoride) and the carrier (chitosan) showed antimicrobial activity against the most important pathogenic bacterial *Streptococcus mutans*. Therefore, nanosilver fluoride systems show the potential to control tooth decay and possibly reduce dental caries.

In the same year, a randomized placebo-controlled clinical trial evaluating the effectiveness of nanosilver fluoride was conducted by dos Santos et al. [58]. One hundred and thirty primary teeth were included in the study and treated with a new formulation of nanosilver fluoride once a year. The concentrations of the components in micrograms per milliliter, were as follows: chitosan 28.585 mg/mL, Ag^+^ 376.5 mg/mL and sodium fluoride 5028.3 mg/mL. The effect of arresting caries development was significant without staining the teeth surface. In the research, the authors have drawn up a detailed protocol for the application of the novel anti-caries agent as part of the individual plan for caries prevention.

There are only a few clinical trials, studying the effect of nanoparticles in the caries prevention treatment for children, which makes them highly informative and useful. Apart from the afore-mentioned article from 2014, in 2017 the results of a randomized, controlled, split-mouth, double-blinded, crossover, and prospective pilot clinical study have been reported [128]. Among twelve children of age between 7 and 8 years, the enamel surface of the permanent incisors and first molars was treated with two types of agents–nano-silver fluoride (NSF) solution (experimental group) and saline solution (control group). Statistically significant lower values of *Streptococcus mutans* levels and colonies were found when enamel was treated with NSF nanoparticles. In addition to these findings, the pH of the biofilm and dental plaque accumulation via the Simplified Oral Hygiene Index (OHI-S) were measured at several time points during the experiment. The authors found that the application of the investigated nanosystem could not affect the acidity of bacterial biofilm, whereas it resulted in a reduction in plaque accumulation.

#### 4.1.3. Calcium Phosphate

Calcium phosphate, usually in the form of amorphous calcium phosphate (ACP), plays a significant role in primary and secondary prevention of dental caries [6]. Zhang et al. investigated the effect of chitosan nanoparticles including ACP for remineralization of enamel subsurface lesions [129]. The scanning electronic microscope observations showed significantly higher efficiency of the remineralizing effect on the enamel surface of prepared ACP-chitosan nanoparticles in comparison with fluoride treatment.

Beside the mineralizing effect of calcium phosphate, it could be successfully used in the coating of polymer microspheres with biomimetic layers [130]. These particles have been investigated as an alternative strategy in biomaterials for their effect on bone and hard dental tissue remineralization. The controlled release of ions resulted in the formation of an apatite layer on the tissue surface.

#### 4.1.4. Other Effective Preventive Agents

In 2014, Ruan et al. evaluated the effect of amelogenin-chitosan nanoparticles included in hydrogel for enamel remineralization [6,131]. Along with the significant improvement of mechanical properties of the treated enamel, the gel demonstrated a suppressive effect on bacterial growth. Thus, in this research two different mechanisms of caries prevention have been found—mineralizing effect of the amelogenin-chitosan gel by regrowth of the apatite crystals as well as inhibition of dental biofilm accumulation. The authors reported a detailed protocol for the application of the hydrogel and emphasized the promising results of its use in future for caries prevention.

### 4.2. Control of Oral Biofilm

Oral biofilm is responsible for the most common oral disease in children—dental caries [132,133,134]. Along with the mineralizing and remineralizing effect of fluoride, prevention of dental caries involves control of oral biofilm. It is considered as one of the most important factors for caries initiation and development [135,136]. Thus, some researchers developed nanosystems directed towards the eradication of the biofilm matrix and resident bacterial microflora. In 2019, Naha et al. synthesized polymer-coated nanoparticles with iron oxide as an active ingredient [136]. The nanoparticles were termed nanozymes (Dex-NZM) and the used polymer was dextran. The results showed a significant reduction in the onset and severity of caries lesions and could be a useful option for alternative treatment of oral disease. However, further studies are necessary to confirm the correlation between biofilm eradication and Dex-NZM.

### 4.3. Endodontic Treatment

The treatment of pulpal inflammation or periapical lesions in permanent teeth with incomplete root development is a tremendous challenge that pediatric dentists are facing when attending dental patients. There are two main methods for endodontic treatment of teeth with necrotic pulp—apexification and revascularisation. Due to the prognostic uncertainties and long-term follow-up of the revascularisation, dentists prefer to using the apexification technique—induction of closure of the apical foramen with mineralized tissue or formation of an artificial apical barrier to allow for condensation of the root filling material and promote an apical seal [137].

Among the numerous techniques and different types of materials, Ca(OH)_2_ necessitating multiple visits for material replacement and mineral trioxide aggregate (MTA) for one-visit apexification are the most frequently used. The application of Ca(OH)_2_ shows numerous advantages and high efficiency in the endodontic treatment in primary and permanent dentition. Due to risk of infection, the trauma of periapical tissues or missed patient appointments, a study conducted by Strom et al. in 2012 reported the same efficiency of Ca^2+^-loaded microspheres structured with a shell composed of alginate [138]. The results of the in vitro experiment demonstrated slow and constant release of ions in the root canal maintaining a pH of about 9. Owing to the advantages of the microspheres in comparison with the commercial formulation of Ca(OH)_2_ paste for root canal filling, the results demonstrated sustained release activity of the Ca^2+^ ions. Additionally, the authors reported suitable size and encapsulation efficiency for application to the root canal of the tooth. Therefore, the application of the Ca-loaded microspheres could be used effectively for a single-visit Ca(OH)_2_ apexification technique.

Thus, the mechanism of action of the sustained drug delivery system could be a useful property in the endodontic treatment of newly erupted permanent teeth with incomplete root development [137,138]. Extensive clinical research is required to further investigate the efficacy of this new treatment approach to overcome the technical and environmental factors.

### 4.4. Periodontal Diseases

Periodontal disease is considered to be the second most common disease in children after dental caries [139,140,141]. Gingival inflammation, known as gingivitis, is typical for children, especially for the age of adolescence [140]. The changes in the periodontium that are caused by gingival inflammation are reversible and the management plan is simple to perform. The severe and advanced form of periodontal disease represents an inflammation of the whole periodontal complex, known as periodontitis, and its development is associated with displacement of the gingival attachment, loss of alveolar bone, periodontal pocket, and gingival recession formation [142,143,144]. In contrast to adults, in pediatric dental patients, aggressive clinical forms of periodontal diseases are prevalent [145,146,147]. Due to the multifactorial etiology and complex pathogenesis as well as the incidence of aggressive periodontal inflammation as a secondary condition of a systemic disease, their treatment is very difficult and complicated. The most common treatment plan includes symptomatic support treatment and monitoring.

Some bacterial species play a significant role in the development of aggressive periodontal inflammation such as *Aggregatibacter actinomycetemcomitans, Porphyromonas gingivalis*, *Prevotella intermedia*. When they are part of the microflora of the gingival sulcus area, antibiotic therapy is needed to accompany the support treatment. The innovative and successful therapeutic approach involves sustained release drug delivery systems, ensuring a controlled drug release from biocompatible and adhesive carriers in the periodontal pocket for a long period of time [148,149]. Recent studies described the effective application of polysaccharide nanoparticles with different active agents in the periodontal treatment [148,149].

#### 4.4.1. Chlorhexidine Gluconate

Chlorhexidine gluconate is widely used as a drug for inflammation of periodontal tissues due to its antiseptic, antifungal, antibacterial, antiviral effect, and high activity against dental biofilm formation [150,151,152]. Several studies investigated the effect of chlorhexidine nanoparticles when applied in the periodontal pocket, included in toothpaste, in gels, etc. [149,153]. Kovtun et al. prepared nanoparticles, based on calcium phosphate and chlorhexidine coated with cellulose, demonstrating both anti-caries and anti-inflammatory activities [153]. The nanosystems showed antibacterial action against *E. coli* and *L. casei* along with the mineralizing effects of calcium and phosphate ions which were firmly attached to the surface of enamel and dentin.

#### 4.4.2. Antibiotics

Along with the broad spectrum of chlorhexidine, tetracycline is considered to be a gold standard in the topical treatment of aggressive periodontal diseases in pediatric dental patients [154,155,156,157]. The efficiency of the antibacterial action of chitosan-based nanoparticles with doxycycline has been studied in 2020 by Xu et al. The findings demonstrated a high level of inhibition of the oral biofilm formation and biocompatibility of the formulation [158]. Polysaccharides have been used for the development of micro- and nanosystems in periodontal treatment encapsulating metronidazole, minocycline, amoxicillin, erythromycin [25,26,64,66].

## 5. Future Perspectives

This review has shown that micro- and nanotechnologies have entered dentistry and may become a promising tool in improving the effectiveness and safety of oral treatment. More and more dental products based on micro- and nanostructures are developed and the strong acceleration in research activities to create a new quality dental materials and therapeutics is expected to continue in the future.

Moreover, the patient-centric approach and the personalized therapy in the treatment of oral diseases are gaining great popularity and are likely to become the focus of future therapeutic dental strategies. These strategies involve drug treatments targeting the main signaling pathway (key receptor or molecule) that initiates the disease process. Due to the incredible progress of science and technology, it is possible to design and apply specific therapeutic agents, according to the individual molecular profile of the patient. With this personalized approach, oral treatment is expected to be more effective and associated with reduced side effects. Therefore, a significant increase in future investment in the development of such therapeutic targeting agents, including novel micro- and nano- drug delivery systems, is anticipated.

Approved medicinal products based on micro- and nanoscale polysaccharide carriers are already available on the pharmaceutical market. However, the use of such therapeutic systems in dental practice is relatively new and there are some uncertainties as to whether these structures can cause long term side effects in the body. Like any pharmaceutical product, micro- and nanomaterials must undergo long and rigorous regulation before being launched on the market, which includes a series of clinical trials. Despite the proven biocompatibility and biodegradability of natural polysaccharides, safety profiles of formulated polymer micro- and nano-sized structures should be subject of mandatory studies, especially when agents with a potential risk of toxicity are used during their production. Most of the studies related to dental micro- and nanomaterials are only in vitro experiments, and the in vivo behavior of the formulations developed was in most cases not investigated or demonstrated. The proposed new dental therapeutics need to be tested in real clinical situations to prove their safety and efficacy. The future application of such innovative therapeutic systems in dentistry requires in-depth preliminary investigation and strict regulation.

Polysaccharides have been extensively studied for their unique characteristics as drug carriers and dental applications ranging from preventive dentistry to bone regeneration in oral surgery. Nevertheless, more research is needed to further characterize these biomaterials and to expand their use effectively in dental treatments.

## 6. Conclusions

The use of polysaccharides as carriers for drug delivery is promising and advantages, such as being non-toxic, biocompatible and biodegradable, makes such systems favorable for dental therapy and further improvement of clinical routine especially in the pediatric practice. The performed literature overview showed that in addition to the widely exploited polymers chitosan and sodium alginate, there are several other promising polysaccharides that can also be successfully included in the development of micro- and nano-sized dental therapeutic systems. By selecting an adequate drug carrier, using an appropriate production method and after carefully studying the influence of the production parameters, particulate drug delivery systems with desired physico-chemical characteristics could be developed with high efficiency and reproducibility. Such systems are not only a new approach to the treatment of various dental diseases, but by providing controlled and/or targeted drug release, they can be a much more successful alternative to conventional therapies. Polysaccharide-based micro- and nano drug delivery systems are relatively new therapeutics in dentistry, which will continue undergoing rapid development in the future, taking their perspective place in the personalized oral treatment.

## Figures and Tables

**Table 1:** Polymer micro- and nanostructures as drug carriers.

Characteristics	Polymer Microstructures	Polymer Nanostructures
Size range	1–1000 µm	1–1000 nm
Preparation methods	Spray-drying Solvent evaporation Ionic gelation Emulsion solvent evaporation Solvent extraction Complex coacervation Polymerization	Nano spray-drying Solvent evaporation Ionic gelation Solvent diffusion Nanoprecipitation Reverse salting-out Polymerization
Types of polymer structures	Microspheres Microcapsules Microbeads Microfilms Microneedles Microchips Microsponges	Nanospheres Nanocapsules Nanogels Nanofibers Nanotubes Micelles Polymersomes
Advantages as drug carriers	Targeted drug delivery Sustained drug release Multiple unit drug delivery Increased drug loading High drug encapsulation efficiency	Targeted drug delivery Sustained drug release Enhanced drug solubility Improved bioavailability Increased cellular uptake Variable administration routes

**Table 2:** Chitosan-based micro- and nanoparticles as drug carriers intended for dental application.

Active Substance	Production Method	Particle Diameter	Entrapment Efficiency	Application	Ref.
Amoxicillin Clavulanic acid	Ionic gelation	45–270 nm	NA*	Bacterial plaque	[52]
Bupivacaine	Emulsion technique	NA*	83%	Dental pain	[53]
Ketoprofen	Spray drying	2–3 µm	54–62%	Periodontitis	[30]
Metronidazole	Emulsion technique	43 μm	59%	Periodontitis	[26]
Metronidazole	External gelation	800 µm	60–75%	Periodontitis	[25]
Miconazole	Complex coacervation	1000 μm	49–67%	Oral candidiasis	[31]
Nal-P-113 peptide	Polymerization	216.20 nm	89%	Root caries restoration; periodontitis	[32]
Ornidazole	Emulsion- ionotropic gelation	29–53 µm	11–32%	Periodontitis	[47]
Sodium fluoride	Ionic gelation	100 nm	4–6%	Caries prevention	[54]
Sodium fluoride	Ionic gelation	219 nm	30%	Caries prevention	[55]
Sodium fluoride	Spray drying	3–6 µm	74–84%	Caries prevention	[56]
Silver fluoride	Reduction of silver nitrate	6 nm	NA *	Caries prevention	[57]
Silver sodium fluoride	Reduction of silver nitrate	3–4 nm	NA *	Caries prevention	[58]
Tetracycline	Gelation technique	1400–1700 μm	NA *	Periodontitis	[48]

* NA—not available information.

## Data Availability

The data presented in this study are available on request from the corresponding author.
